# Common Carotid Artery Occlusion: A Case Series

**DOI:** 10.1155/2013/198595

**Published:** 2013-09-16

**Authors:** Zoltán Bajkó, Rodica Bălaşa, Anca Moţăţăianu, Smaranda Maier, Octavia Claudia Chebuţ, Szabolcs Szatmári

**Affiliations:** Department of Neurology, Neurology Clinic, Mureş County Clinical Emergency Hospital, University of Medicine and Pharmacy Târgu Mureş, Marinescu Gh. Street 50, 540136 Mureş County, Romania

## Abstract

*Subjects and Methods*. We analysed 5000 cerebrovascular ultrasound records. A total of 0.4% of the patients had common carotid artery occlusion (CCAO). *Results*. The mean age was 59.8 ± 14.2 years, and the male/female ratio was 2.33. The most frequent risk factors were hypertension, ischaemic heart disease, dyslipidemia, diabetes mellitus, and smoking. Right-sided and left-sided CCAO occurred in 65% and 30% of the cases, respectively, and bilateral occlusion was detected in one case (5%). Patent bifurcation was observed in 10 cases of CCAO in which the anterograde flow in the ICA was maintained from the external carotid artery with reversed flow. In two of the cases, the occluded CCA was hypoplastic. The aetiology of CCAO in the majority of cases was the atherosclerosis (15 cases). The male/female ratio was higher in the patients with occluded distal vessels, and the short-term outcome was poorer. Only two cases from this series underwent revascularisation surgery. Spontaneous recanalisation was observed in one case. *Conclusions*. The most frequent cause of CCAO was atherosclerosis. The outcome is improved in the cases with patent distal vessels, and spontaneous recanalisation is possible. Treatment methods have not been standardised. Surgical revascularisation is possible in cases of patent distal vessels, but the indications are debatable.

## 1. Introduction

Common carotid artery (CCA) occlusion is a rare cause of cerebrovascular events. The prevalence is approximately 0.24–5% in stroke patients [1–6].

In contrast to the large amount of data in the literature about internal carotid artery occlusion, there is little information regarding the incidence, clinical presentation, ultrasound findings, haemodynamics, causes, and treatment of common carotid artery occlusion (CCAO).

CCA occlusion is generally associated with occlusion of the distal vessels (internal carotid artery (ICAs) and external carotid artery (ECA)). In some cases, blood flow in the ICA and ECA is maintained by collateral circulation via extracranial branches through the retrograde external carotid artery. Recognising the patency of the distal vessels is important because it may allow for effective surgical revascularisation [5]. 

Doppler ultrasound examination is an important tool in the diagnosis of CCAO.

The *aim* of this study was to discuss the clinical findings, ultrasonographic characteristics, possible mechanisms, and treatment possibilities of common carotid artery occlusion. 

## 2. Subjects and Methods

We analysed 5000 duplex cerebrovascular ultrasound records performed during a 5-year period from 2008 to 2012 from patients who were examined in the Ultrasound Laboratory of Neurology Clinic I at Mureş County Clinical Emergency Hospital. The examinations were performed using a Siemens Acuson Antares ultrasound system (VFX13-5 MHz linear transducer, PX4-1 MHz transcranial transducer, CW5 Doppler pencil transducer). In questionable cases, the ultrasound findings were confirmed by angiography or CT angiography. 

The duplex criteria for carotid artery occlusion included increased echogenicity throughout the course of the vessel, lack of cross-sectional pulsation, and absence of flow signal [2].

A total of 160 out of 5000 (3.2%) patients with cerebro-vascular disease who were referred for carotid sonography had a carotid artery occlusion. Of these patients, 20 (0.4%) presented with CCA occlusion or CCA occlusion associated with ICA occlusion. The medical history, risk factors, and clinical characteristics of these cases were obtained from the archived medical records. 

We attempted to determine the possible causes of CCAO from the medical history, clinical data, ultrasonographic findings, and laboratory results in all of the cases. Atherosclerotic aetiology was suspected when the patient presented with vascular risk factors, and the vascular ultrasound findings were suggestive of atherosclerosis (e.g., atherosclerotic vessel wall changes in the extracranial arteries and the CCA filled with heterogeneous or hyperechogenic atherothrombotic material). Takayasu's arteritis was suspected if the patient met the 1990 American College of Rheumatology diagnostic criteria [7]. Postirradiation arteriopathy was diagnosed if the patients presented with a history of radiotherapy in the neck region due to malignancy. Cardiac embolism was suspected if the patients had cardiac arrhythmia or other cardiac disease with a high risk of embolism without signs of atherosclerosis. Primary or secondary hypercoagulability was suspected in young patients with a CCA occlusion without signs of atherosclerosis or embolic heart disease and who did not fulfil the criterion for Takayasu's arteritis, were not irradiated in the neck, and presented with other thrombotic events. In cases of occlusion with a cardioembolic origin or in situ thrombosis, the vessel lumen is filled with hypoechogenic thrombotic material.

Patients were considered to have asymptomatic common carotid artery occlusion if there were no reports of symptoms or signs attributable to vascular events in the region of the occluded CCA. Symptomatic CCAO was defined as the occurrence of at least one transient ischaemic attack (TIA) or stroke attributable to carotid circulation on the same side prompting ultrasonography that showed no patency of the corresponding CCA [8].

A major stroke was defined as a combination of a disturbance of consciousness and at least two of the following neurological signs: conjugate deviation, homonymous hemianopsia, aphasia, and hemiplegia. Other events were considered minor strokes [2]. 

Severity in patients with acute stroke was assessed using the National Institutes of Health Stroke Scale (NIHSS) [9] and the short-term stroke outcome based on the Glasgow Outcome Scale (GOS) (1-dead, 2-vegetative state, 3-severely disabled, 4-moderately disabled, and 5-good recovery) [10].

## 3. Results

The 20 cases of CCA occlusion represented 0.4% of all of the patients with cerebro-vascular pathology who underwent cervicocerebral duplex ultrasound examination. 

Table 1 shows the demographic characteristics, risk factors, and indications for ultrasound examination. The mean age of the patients with CCAO was 59.8 ± 14.2 years (min. 26, max. 86), and the male/female ratio was 2.33. The most frequent risk factors were hypertension, ischaemic heart disease, dyslipidemia, diabetes mellitus, and smoking. The indication for ultrasound examination was an acute stroke or poststroke state in the majority of the cases. 

The diagnosis of CCAO was established based on ultrasound (US) examination. The US findings were confirmed with CT angiography in 2 cases and digital subtraction angiography (DSA) in 3 cases.

The right and left sides of the CCA were occluded in 65% (13/20) and 30% (6/20) of the cases, respectively, and the CCA was occluded bilaterally in one case (5%). 

**Figure 1:**
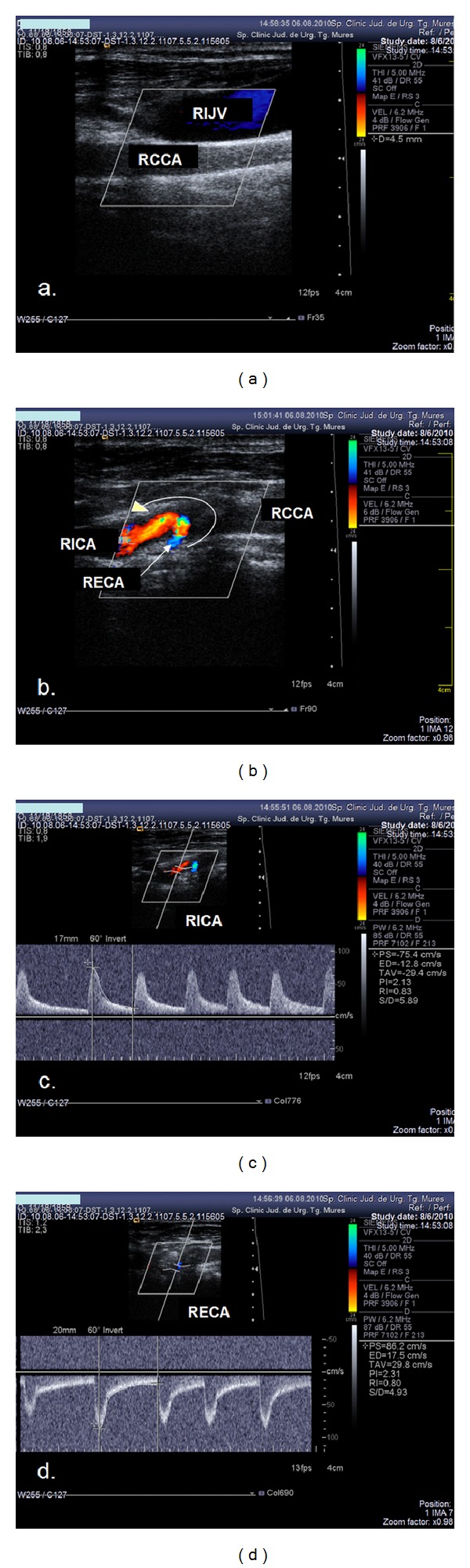
Ultrasound examination showing CCA occlusion with patent distal vessels. (a) Colour mode examination: no flow in the right common carotid artery, and the vessel lumen is filled with thrombotic material. (b) Colour mode examination of carotid bifurcation: reversed flow in the ECA, anterograde flow in the ICA. (c) Duplex mode examination: anterograde flow in the ICA. (d) Duplex mode examination: retrograde flow in the ECA.

**Figure 2:**
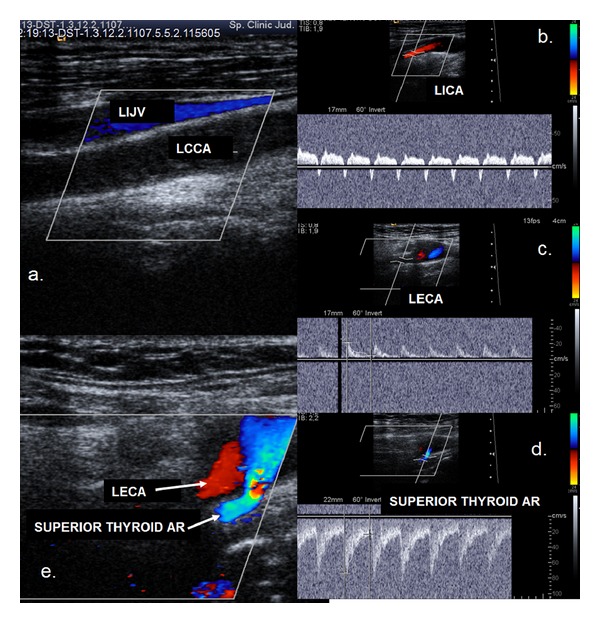
Ultrasound examination showing common carotid artery occlusion with patent distal vessels. Anterograde flow in both the ICA and ECA. (a) Colour mode examination: no flow in the left common carotid artery (LCCA), and the vessel lumen is filled with thrombotic material. (b) Duplex mode examination: anterograde flow in the left internal carotid artery with a steal effect, deceleration, and inversed flow in mesosystole. (c) Duplex mode examination: anterograde flow in the left external carotid artery. (d) Duplex mode examination: retrograde flow in the left superior thyroid artery. (e) Colour mode examination: retrograde flow in the left superior thyroid artery and anterograde flow in LECA.

A patent bifurcation was detected in 10 of the cases of CCAO (50%). In 9 of these cases, the anterograde flow in the ICA was maintained from the external carotid artery with a reversed flow direction (Figure 1). In one case, both the ICA and ECA presented with anterograde flow, and the flow was reversed only in the first branch of the ECA (superior thyroid artery). This high retrograde flow supplied both the ECA and ICA. Interestingly, the ECA exerted an incomplete steal effect on the ICA (deceleration and short negative flow in mesosystole on the spectral waveform of the ICA) (Figure 2).

The occluded CCA was hypoplastic in two of the cases (Figure 4).

**Figure 3:**
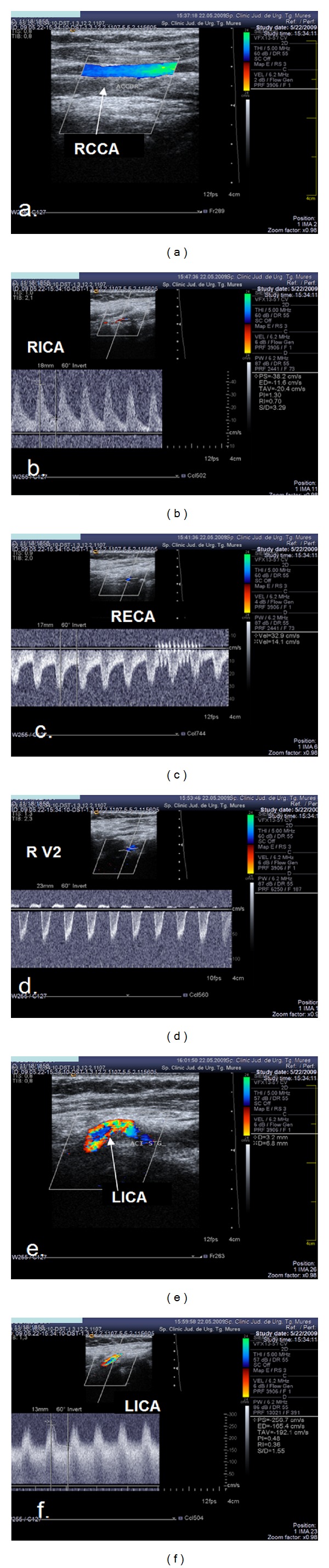
Ultrasound examination showing multiple steno-occlusive lesions in the cervical vessels. (a) Colour mode examination: RCCA occlusion, no flow in the right common carotid artery, and the vessel lumen is filled with thrombotic material. (b), (c) Duplex mode examination: anterograde flow in the right ICA and retrograde flow in the right ECA. (d) Duplex mode examination, subclavian steal phenomenon, and retrograde flow in the right vertebral artery. (e), (f) Colour mode and duplex mode examination and severe left internal carotid artery stenosis.

**Figure 4:**
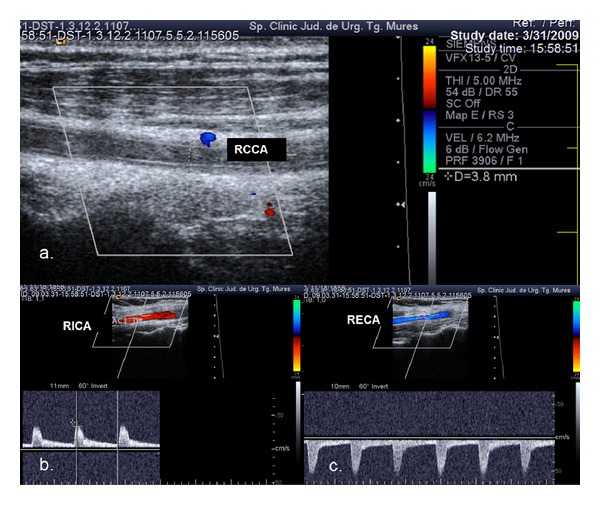
Ultrasound examination showing hypoplastic and occluded right CCA. (a) Colour mode examination: RCCA occlusion, no flow in the right common carotid artery, the vessel lumen is filled with thrombotic material, and the vessel diameter is 3.8 mm. (b), (c) Duplex mode examination: anterograde flow in the right ICA and retrograde flow in the right ECA.

In 9 cases, severe contralateral CCA or ICA stenosis or occlusion was observed, and a subclavian steal phenomenon or severe vertebral artery stenosis was shown in 3 cases.  Figure 3 shows the ultrasonographic changes in a case with multiple cervical vascular pathology, including RCCA occlusion with patent distal vessels, and right subclavian steal phenomenon and significant LICA stenosis (Figure 3).

The CCA occlusion was symptomatic (at least one transient ischaemic attack or stroke attributable to carotid circulation ipsilateral to CCA occlusion) in 9 cases and asymptomatic (no reports of symptoms or signs attributable to vascular events in the region of the occluded CCA) in 11 cases. In the symptomatic group, 6 cases presented with minor stroke or TIA, and 3 cases presented with a major stroke. In the asymptomatic group, 4 of the patients did not have ischaemic stroke with arterial origin but presented with nonspecific symptomatology, such as dizziness (2 cases), or the CCAO was an accidental finding during a routine ultrasound examination for other cerebral pathologies (case 13 presented with aneurysmal subarachnoid haemorrhage and case 20 presented with deep cerebral venous thrombosis) (Table 1). A total of 7 patients in the asymptomatic group presented with strokes that affected the cerebral hemisphere contralateral to the CCAO (4 minor strokes, 3 major strokes). 

In 15 of the cases, the aetiology of CCAO was atherosclerosis. One patient fulfilled the American College of Rheumatology 1990 diagnostic criteria [7] for Takayasu's arteritis, one case presented with neck irradiation for laryngeal cancer in their medical history, one case had a likely aetiology of cardioembolism (paroxysmal atrial fibrillation), and 2 cases had hypercoagulability (case 10 developed CCAO after surgical intervention for free floating thrombus removal from the right carotid bulb, and the CCAO finding was incidental in case 20 with deep cerebral vein thrombosis). 

There was no significant difference between the patients with patent distal vessels and occluded distal vessels regarding age and vascular risk factors. In the patients with occluded distal vessels, the systolic blood pressure was significantly higher, the NIHSS was significantly higher, and GOS was significantly lower (Table 2). The male/female ratio was higher in the patients with occluded distal vessels. 

Only two cases underwent revascularisation surgery. One patient died after the intervention (developed a major stroke), and the second patient was asymptomatic after surgery. Spontaneous recanalisation of the occluded CCA was observed in one case. 

## 4. Discussion

The clinical presentation of CCA occlusion ranges from asymptomatic to severe cerebrovascular events. Because the asymptomatic cases are diagnosed accidentally, the actual incidence of CCA occlusion is not known. 

Parthenis et al. reported a 0.54% prevalence of CCAO and 2.8% of ICAO based on a large ultrasonographic database of 6415 cases [11]. Chang reported a 0.24% prevalence of CCAO and 2.5% of ICAO [2]. Other authors have reported a prevalence ranging from 1 to 5% in patients with stroke [2, 12–14]. These results are consistent with the data in this study (3.2% prevalence for ICA occlusion and 0.4% for CCA occlusion).

The mechanisms behind thrombotic processes may differ according to aetiology. In patients with atherosclerotic occlusion, the thrombosis may be retrograde because atherosclerosis most frequently affects carotid bifurcation. In patients with Takayasu's arteritis, the thrombotic process may be anterograde, with more frequent involvement of the aortic arch, subclavian arteries, and brachiocephalic trunk. 

CCA occlusion can be classified based on the patency of the distal vessels. In Type I occlusion, there is a complete occlusion of both the CCA and the ICA. In Type II occlusion, the distal vessels are patent [15]. In Type I occlusion, ischemic events occur more frequently, suggesting a haemodynamic aetiology. In Type II occlusion, the ICA is supplied by collateral blood flow via extracranial branches through the retrograde external carotid artery. In these cases, the ischaemic events are caused by artery-to-artery embolisms [6, 15, 16].

Parthenis at al. [11] proposed the following detailed classifications: Type I: isolated CCAO with patent distal vessels; Type Ia: flow direction from the ECA to ICA; Type Ib: flow direction from the ICA to ECA; Types II and III: isolated patency of the ECA and ICA; and Type IV: concomitant occlusion of both distal vessels. According to this classification, the majority of the cases in this study can be categorised in Type Ia and Type IV. We did not determine any cases with patent distal vessels presenting with a flow direction from ICA to ECA or cases with isolated ICA or ECA patency in this study. 

In our series, male predominance (M/F = 2.33) was evident, which is similar to the data in the literature [2, 4, 13, 14, 17]. In contrast, the right sided ICA was more frequently affected in our series (65% of cases). Chang et al. and previous studies have reported a higher prevalence of left sided involvement [2, 12, 13, 17]. Parthenis et al. reported an equal distribution between the right and left sides [11]. Bilateral CCA occlusion is rare and has primarily been reported in cases of Takayasu's arteritis and rare cases in patients with atherosclerosis [18, 19]. An 86-year-old male patient in our study with bilateral occlusion and widespread atherosclerosis without focal neurological signs was examined by ultrasound for nonspecific dizziness. 

The risk factor profile did not differ from previously published data. Chang et al. and Collice at al. reported a high prevalence of hypertension and heart disease [2, 13]. In our patient group, the prevalence of hypertension was 90%, and the prevalence of ischaemic heart disease was 75%. 

In the majority of published case series, the major cause of CCAO is atherosclerosis [1, 2, 6]. A higher prevalence of Takayasu's arteritis was reported in the Asian population [18]. Rarer causes include postirradiation arteriopathy, cardiac embolism, dissection of the aortic arch and CCA, aortic arch aneurysm, hypercoagulability, fibromuscular dysplasia, and craniocervical traumatism [2, 20]. In the majority of our cases (75%), the aetiology of CCA was atherosclerosis. 

There is no data in the literature related to CCA hypoplasia as a predisposing factor for occlusion. Two of the cases from our series presented with a CCA diameter less than 4 mm. 

In cases of CCAO, perfusion of the ipsilateral cerebral hemisphere is provided through collateral circulation. The extracranial collateral vessels fill the ECA in a retrograde manner and maintain the anterograde flow in the ICA. The extracranial collateral flow originates from the ipsilateral subclavian artery via the costocervical or thyrocervical trunks and the vertebral artery and, to a lesser degree, from the contralateral ECA through the superior thyroid and lingual, facial, and occipital branches [6, 11, 20]. The flow is maintained intracranially through the circle of Willis via the anterior and posterior communicating arteries. We did not find any data similar to our case (Figure 2) in the literature where both the ICA and ECA had anterograde flow and were supplied through a major branch of the ECA with retrograde flow. 

A total of 16 patients in our series presented with a cerebro-vascular event, 6 minor strokes or TIA and 3 major strokes affecting the ipsilateral hemisphere and 4 minor strokes or TIA and 3 major strokes affecting the contralateral cerebral hemisphere. In the case series by Chang et al., the occurrence of stroke was more common than TIA, and most of the strokes were major. Other case series reported more frequent TIAs [4, 14, 17, 20]. Parthenis et al. reported frequent positional-related symptoms. In some cases, TIAs affected the contralateral hemisphere to the CCAO. 

Similar to our results, the majority of case series has reported milder neurological symptoms and more favourable outcomes in patients with patent distal vessels. Zbornikova and Lassvik published a series of 21 patients with CCAO and found 12 cases of patent bifurcation. In 10 of the cases, flow was maintained from the ECA to the ICA, and the flow direction was opposite (from ICA to ECA) in the remaining 2 cases. Although most of the patients with patent bifurcation presented with amaurosis fugax and vertigo attacks, none of the patients with patent distal vessels and well-functioning intracranial collaterals had a major stroke. In contrast, 50% of the patients with occluded distal vessels presented with a major stroke [21].

Arteriography remains the gold standard for accurately diagnosing carotid artery stenoocclusive disease. However, noninvasive duplex sonography, CT angiography, and MR angiography have partially replaced arteriography in daily clinical practice. The accuracy of duplex sonography should be much higher in the diagnosis of CCAO compared to ICA occlusion due to accessibility of the CCA [2]. Arteriography has several limitations in cases of completely occluded CCA because of inadequate concentrations of contrast medium, variable collateral circulation, minimal flow in the distal branches, and poor delayed images [2, 11]. In the case series reported by Parthenis et al., Doppler ultrasound examination showed at least 1 patent distal vessel in 29 out of 35 cases, and arteriography failed to demonstrate patency of the distal vessels in 19 of the patients [11]. Colour Doppler with low PRF allows for easy identification of the low flow states in the vessels distal to the occluded CCA [2, 5, 6, 11, 22]. The ultrasonographic features of the intraluminal thrombotic material allow us to draw conclusions regarding the aetiology of the occlusion and, in some cases, the age of the occlusion. 

Most clinicians have concluded that colour flow duplex examination is the hallmark of detecting a patent ICA despite CCA occlusion [23].

Several very rare cases with spontaneous recanalisation of the occluded CCA have been reported in the literature. Ultrasound follow-up examinations are useful to assess recanalisation [24]. Shah reported a case of CCAO with spontaneous recanalisation and subsequent embolic middle cerebral artery occlusion [25]. 

There are little data in the literature regarding the various treatment strategies for CCAO. Successful revascularisation is dependent on the state of the distal branches. As a result, establishing patency of the ICA and ECA is essential prior to an intervention. Several small series reported in the literature have shown excellent revascularisation results in relieving the symptoms of cerebral ischaemia.

Martin et al. reported a series of 8 cases of CCAO that were surgically treated (bypass with the saphenous vein to either the carotid bifurcation, the internal carotid artery, or the external carotid artery). That study established the following indications for surgical treatment: ipsilateral TIA, recent nondisabling hemispheric stroke, and transient nonhemispheric cerebral symptoms or prophylactic revascularisation before major surgical interventions (planned aortic surgery). There were no perioperative strokes, occlusions, or deaths [26]. The natural history of CCAO with patent ICA in asymptomatic cases is not known [22]. The surgical interventions are bypass procedures (subclavian to the CCA, subclavian to the ICA, subclavian to the ECA, axillary to the CCA, or ascending aortic bifurcation graft to the CCA) or endarterectomy [2].

Pintér et al. reported a patient with CCA occlusion who underwent a hybrid treatment consisting of endarterectomy plus stenting [27]. An eversion carotid bifurcation endarterectomy was performed with a fluoroscopically guided retrograde ring-stripper common carotid endarterectomy and stenting of the residual stenosis in the disobliterated artery. 

Sharma et al. reported 3 cases of thrombotic CCA occlusion associated with acute ischaemic stroke due to tandem occlusion in the intracranial arteries that were treated with an intravenous tissue plasminogen activator. In 2 out of 3 cases, there was marked early neurological improvement [28].

Because there are no separate evidence-based recommendations for atherosclerotic CCA occlusion, the general recommendations for managing patients with atherosclerotic carotid occlusion (including atherosclerotic CCAOs) are valid in these rare cases. Patients with acute ischaemic stroke due to atherosclerotic carotid artery occlusion (ACAO) should receive intravenous tissue plasminogen activator if they meet the eligibility criteria. Patients who are not eligible for intravenous tissue plasminogen activator should receive aspirin because heparin and heparin-like drugs do not improve the outcome. Therapy to prevent recurrent stroke in patients with ACAO should consist of lifestyle modifications, risk factor intervention, and antiplatelet drugs. Warfarin is not indicated, and surgical or endovascular procedures have not been shown to be effective in treating or preventing stroke due to ACAO. Asymptomatic carotid occlusion has a benign prognosis and requires no specific treatment other than lifestyle modification and risk factor intervention [29].

## 5. Conclusions

The most frequent cause of CCAO is atherosclerosis. Duplex sonography is an important noninvasive, reliable method for diagnosing CCA occlusion and establishing the patency of distal vessels. The outcome is improved in cases with patent distal vessels. In rare cases, spontaneous recanalisation is possible. Due to the low incidence of CCAO, treatment methods have not been standardised. In cases of patent distal vessels, surgical revascularisation is possible, but the indications for surgery are debatable.

## Figures and Tables

**Table 1:** Demographic data, risk factors, and indications for ultrasound examination in patients with CCA occlusion.

Patient	Age	Sex	Vascular risk factors	Indication for ultrasound examination
(1)	55	F	HT	Acute stroke
(2)	57	M	HT, IHD	TIA, poststroke state
(3)	76	M	HT, IHD, DM, and PVD	Acute stroke
(4)	60	M	HT, IHD, DL, and smoking	Acute stroke
(5)	49	M	HT, smoking, and alcohol	Acute stroke
(6)	60	M	HT, IHD, PVD, and DM	Poststroke state
(7)	50	F	HT, IHD, and DL	Poststroke state
(8)	67	M	HT, IHD, DM, DL, and obesity	Acute stroke
(9)	55	F	HT, IHD, DM, and DL	Dizziness, headache
(10)	26	M	Smoking	Acute stroke
(11)	70	M	HT, IHD, DL, and PVD	Acute stroke
(12)	74	M	HT, IHD, and PVD	Acute stroke
(13)	37	M	Smoking, AF	Subarachnoid haemorrhage
(14)	86	M	HT, IHD, PVD, and AF	Dizziness
(15)	60	F	HT, IHD, PVD, DL, and smoking	Acute stroke
(16)	74	M	HT, IHD, and PVD	Acute stroke
(17)	70	F	HT, IHD, and DL	Dizziness
(18)	59	F	HT, IHD, and DL	Acute stroke
(19)	67	M	HT, IHD, and AF	Acute stroke
(20)	44	M	HT, smoking	Venous infarction

	Mean ± SD: 59.8 ± 14.2	M/F: 2.33		

Abbreviations: TIA: transient ischaemic attack; HT: hypertension, DL: dyslipidemia; IHD: ischaemic heart disease; DM: diabetes mellitus; PVD: peripheral vascular disease; AF: atrial fibrillation.

**Table 2:** Demographic data, blood pressure, and stroke scales in the patients with occluded and patent distal vessels.

	Age	Sex (M/F)	SBP	DBP	NIHSS	GOS
Patients with occluded distal vessels	58.6 ± 14.8	8/2	178.6 ± 28.7	94.2 ± 19.3	10.3 ± 5.12	3.5 ± 0.72
Patients with patent distal vessels	61 ± 14.1	6/4	142.5 ± 24.3	79.3 ± 11.4	3.5 ± 2.9	4.3 ± 0.48
*P* value	NS		0.017	NS	0.007	0.009

Abbreviations: SBP: systolic blood pressure; DBP: diastolic blood pressure; NIHSS: National Institutes of Health Stroke Scale; GOS: Glasgow Outcome Scale.
